# Optimal training strategy for body weight support treadmill training to enhance lower limb motor function and activity of daily living in persons with stroke: a systematic review and meta-analysis of randomized controlled trials

**DOI:** 10.3389/fneur.2025.1649246

**Published:** 2025-09-02

**Authors:** Yan Shi, Junying Liu, Xinxin Zhang

**Affiliations:** Department of Exercise Science, School of Physical Education, Shaanxi Normal University, Xi’an, China

**Keywords:** body weight support treadmill training, stroke, lower limb motor function, activity of daily living, meta-analysis

## Abstract

**Objective:**

This meta-analysis aimed to investigate the effect of body weight support treadmill training (BWSTT) on lower limb motor function and daily living activities in a person with a stroke while also exploring the optimal training strategy.

**Methods:**

Six databases (PubMed, Web of Science, The Cochrane Library, CNKI, Wanfang, and SinoMed) were searched up to August 2025. Randomized controlled trials involving persons with stroke, BWSTT, and outcomes measured by the Fugl-Meyer assessment of lower extremity and Barthel Index scores were included. The risk of bias was assessed using the RoB-2 tool of the Cochrane Collaboration, and the certainty of evidence was assessed using the GRADE tool.

**Results:**

25 studies with 1,749 people with stroke were incorporated into the meta-analysis. The meta-analysis demonstrated that BWSTT significantly outperformed the control group in improving both the Fugl-Meyer lower extremity score (MD = 4.80, 95% CI: 2.90–6.71, *p* < 0.001) and Barthel Index score (MD = 10.53, 95% CI: 7.61–13.46, *p* < 0.001). The certainty of evidence was rated as “very low.” The most effective interventions were observed in persons with a disease duration of 3–6 months (Fugl-Meyer: MD = 4.72, 95% CI: 1.54–7.89, *p* = 0.004; Barthel: MD = 17.58, 95% CI: 11.75–23.40, *p* < 0.001), intervention time of 4–8 weeks (Fugl-Meyer: MD = 5.78, 95% CI: 3.80–7.76, *p* < 0.001; Barthel: MD = 12.85, 95% CI: 3.84–21.87, *p* = 0.005), body weight support over 30% (Fugl-Meyer: MD = 4.51, 95% CI: 1.75–7.28, *p* = 0.001; Barthel: MD = 10.79, 95% CI: 6.91–14.67, *p* < 0.001), and gait speeds of 0.2 m/s or higher (Fugl-Meyer: MD = 4.01, 95% CI: 1.62–6.40, *p* = 0.001; Barthel: MD = 10.61, 95% CI: 1.13–20.10, *p* = 0.03).

**Conclusion:**

BWSTT improved the lower limb function and daily activities of persons with stroke, with optimal outcomes at disease duration of 3–6 months or undergoing interventions for 4–8 weeks, and more than 30% of the maximum body weight support level or using a gait speed exceeding 0.2 m/s. It is unclear whether persons with disease durations of 3–6 months could achieve the same outcomes as those undergoing 4–8 weeks of intervention. The very low quality of evidence suggests that the conclusions require further validation through high-quality randomized controlled trials.

**Systematic review registration:**

http://www.crd.york.ac.uk/PROSPERO/, identifier: CRD42023486562.

## 1 Introduction

Stroke ranks as the second primary cause of mortality worldwide (1). The principal aim of early rehabilitation for a person with a stroke is to recover lower limb motor function to enhance their self-care capabilities (2, 3). Research indicates that more than 80% of people with stroke suffer from lower limb motor impairment, with a considerable number restricted to minimal movement within their residences and unable to participate in social activities (4). This restriction significantly diminishes a person’s quality of life and imposes a considerable psychological burden. As such, identifying effective intervention strategies to assist persons with stroke in recovering their lower limb motor function and daily living activities is a critical challenge in the field of rehabilitation (5). Unfortunately, despite numerous studies on rehabilitation, the best strategy to help people recover lower limb motor function and activity of daily living is still unclear.

Body weight support treadmill training (BWSTT) is a crucial method in early stroke rehabilitation, aiding in weight reduction through overhead harness or pneumatic techniques to facilitate exercise, thereby promoting quicker recovery (3). In recent years, BWSTT has been widely adopted for stroke rehabilitation and has demonstrated considerable efficacy (6–8). Prior research has established that BWSTT significantly enhances lower limb motor function, self-care capabilities, and overall quality of life, facilitating people’s reintegration into familial and societal contexts (9–11). A recent study by Jiang examined the impact of BWSTT on balance and walking ability in persons with stroke, revealing that disease duration and training parameters (including intervention time and load) significantly influenced rehabilitation outcomes (3). Nonetheless, previous studies have mainly focused on the efficacy of BWSTT in improving walking and balance abilities of stroke patients. There is a lack of comprehensive systematic reviews or meta-analyses focusing on lower limb motor function and daily activities.

Therefore, this study aimed to systematically and quantitatively assess the effects of BWSTT on lower limb motor function and activities of daily living in persons with stroke and to explore the optimal intervention strategies to provide a reference for optimizing the effectiveness of clinical interventions.

## 2 Methods

### 2.1 Protocol and registration

This meta-analysis adhered to the Preferred Reporting Items for Systematic Reviews and Meta-Analyses (PRISMA) guidelines (12), and the study protocol is registered in the International Prospective Register of Systematic Reviews database (identifier: CRD42023486562).

### 2.2 Information sources and search strategy

The PubMed, Web of Science, The Cochrane Library, CNKI, Wanfang, and Chinese SinoMed Databases were searched through inception to August 1, 2025. Search terms used included “stroke,” “cerebral vascular accident,” “antigravity treadmill,” “body weight support,” and their respective synonyms. Using the Cochrane Library database as a case study, the precise search approach is outlined as follows:

#1 MeSH descriptor: [Stroke] explode all trees.
#2 Cerebral stroke.
#3 Cerebral vascular accident.
#4 CVA.
#5 #1 OR #2 OR #3 OR #4.
#6 Antigravity treadmill.
#7 Body weight support.
#8 #6 OR #7.
#9 #5 AND #8 in Trials.

### 2.3 Eligibility criteria

Inclusion criteria: (1) Type of study: Randomized Controlled Trial (RCT); (2) Study participants: person with the clinical diagnosis of stroke; (3) Interventions: the experimental group received BWSTT combined with usual rehabilitation, while the control group received only usual rehabilitation; (4) Outcome indexes: Fugl-Meyer assessment of lower extremity score and Barthel Index score. The Fugl-Meyer assessment of the lower extremity and the Barthel Index are two of the most commonly used methods for evaluating lower limb motor function and activity of daily living. The results of previous studies have revealed that they have good reliability for detecting changes over time for persons after stroke rehabilitation and are suitable as outcomes for stroke research and practice (13, 14).

Exclusion criteria: (1) Studies that did not involve BWSTT; (2) Studies with missing or inconsistent outcome measures; (3) Conference abstracts and dissertation papers; and (4) Duplicated publications by the same research team using the same study at different points.

### 2.4 Study selection and data extraction

EndNote X9 software was utilized to eliminate duplicate literature during the study selection process. Two coauthors (YS and JL) independently evaluated the titles and abstracts of the retrieved records utilizing a double-blind methodology, adhering to the established inclusion and exclusion criteria. Full texts of studies that potentially met the inclusion criteria were downloaded for further screening. In cases of disagreement between the two coauthors, a third coauthor (XZ) participated in a joint discussion to determine whether to include the study.

Two coauthors (YS and JL) separately extracted data from the selected studies utilizing a pre-designed form throughout the data extraction process. The extracted data comprised: (1) fundamental study information: first author, year of publication; (2) participant details: sample size, age, disease duration; (3) intervention parameters: intervention time, frequency, body weight support level, and gait speed; (4) baseline and endpoint outcome data (test results before the start of the intervention and test results after the last intervention).

### 2.5 Risk of Bias assessment

The risk of bias for the selected studies was evaluated utilizing the RoB-2 tool of the Cochrane Collaboration (15). The assessment comprised six primary components: (1) Randomization process, (2) Deviations from intended interventions, (3) Missing outcome data, (4) Measurement of the outcome, (5) Selection of the reported result, and (6) Overall. The process of evaluating the quality of the literature was carried out independently by two coauthors (YS and JL). In case of disagreement, a third coauthor (XZ) was added to discuss the matter until a consensus was reached.

### 2.6 Data synthesis

Meta-analyses were performed for the pre-post change outcomes in the intervention and control groups using Review Manager version 5.4. All outcome parameters of the studies included in this meta-analysis were continuous variables, and the same outcome was measured in the same way, so mean differences (MD) with 95% confidence intervals (CI) were used as pooled effect sizes (16). The Chi^2^ test and I^2^ statistic were utilized to evaluate heterogeneity, and a random-effects model was employed when significant heterogeneity was detected (I^2^ > 50%; *p*<0.05). Otherwise, a fixed-effects model was employed (17). Sensitivity analyses were performed by removing studies one by one on heterogeneous outcomes to evaluate the robustness of the findings (3). Subgroup analyses were conducted according to person characteristics and training parameters to determine optimal training strategies, while meta-regression analyses were utilized to investigate sources of heterogeneity (18). Publication bias was tested by drawing funnel plots using Stata 14.0 software and further quantified using Egger’s test. When significant publication bias existed, the effect of publication bias on the results of Meta-analysis was further analyzed by the Duvaland Tweedie trim and fill method (19). A *p*-value less than 0.05 was deemed statistically significant.

### 2.7 Grading of evidence

Two authors (YS and XZ) evaluated the grading of evidence for the Fugl-Meyer assessment of lower extremity score and Barthel Index score outcomes according to the GRADEpro Guideline Development Tool (20). The evaluations were categorized into risk of bias, inconsistency, indirectness, imprecision, and publication bias. Each domain was categorized as “not serious, ““serious,” or “very serious” according to the evaluation criteria, and the overall certainty of the evidence was categorized into four grades: very low, low, moderate, or high.

## 3 Results

### 3.1 Study selection

**Figure 1:**
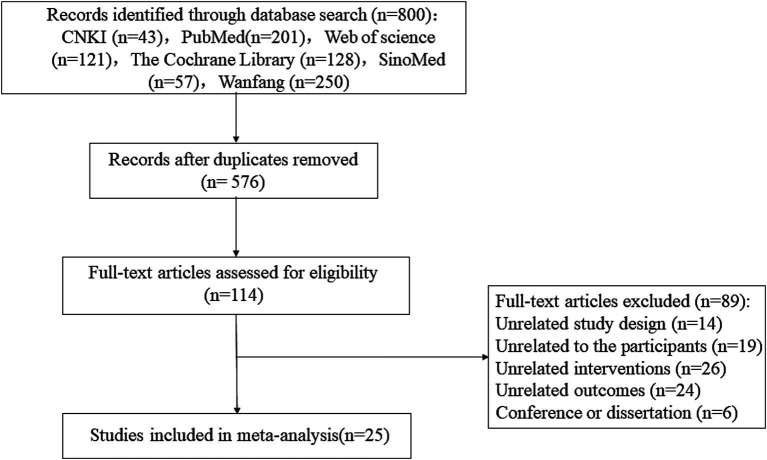
PRISMA flowchart for the study selection process.

The initial database search produced 800 records. Following the removal of duplicates via EndNote, 576 records were retained. After evaluating titles and abstracts, 462 studies deemed irrelevant were excluded. One hundred fourteen records were subjected to full-text screening, excluding 89 studies that failed to meet the inclusion criteria. A total of 25 studies were incorporated into the meta-analysis (9–11, 21–42). Figure 1 illustrates the PRISMA flowchart for the study selection process.

### 3.2 Methodological quality assessment

During the RoB-2 tool assessment, each study was categorized as “low risk,” “some concerns” or “high risk.” If all components were assessed as “low risk,” the overall risk of study bias was defined as low risk; if one or more components were assessed as “some concerns” risk, the overall risk of study bias was defined as “some concerns”; if any one component was categorized as “high risk,” the overall risk of study bias was defined as “high risk” (15). A risk of bias assessment of the 25 included studies showed that five studies (21, 25–28) were considered low risk of bias because no bias in the assessment was detected. Twenty studies (9–11, 22–24, 29–42) were rated at high risk of bias, mainly due to deviation from the intended intervention. In addition, one study (9) observed uncertainty in the measurement of the outcome and the missing data on the outcome. Detailed assessment result is presented in Supplementary Figure S1.

### 3.3 Descriptive characteristics of included studies

The 25 studies included in this meta-analysis involved 1,749 people, most of whom were middle-aged or older adults. The shortest disease duration among the people was 15 days, while the longest was 50 months. In terms of support mechanisms, most studies used overhead harnesses. Regarding intervention time, the shortest duration was 3 weeks, and the longest was 4 months. Regarding body weight support, the minimum level was 8%, while the maximum ranged from 65 to 100%. For outcomes, 20 studies assessed the Fugl-Meyer assessment of lower extremity score, and 16 evaluated the Barthel Index score. In terms of usual rehabilitation training, the Normal limb Position, Acupuncture, PNF, Stand-up, Balance, Gait, Muscle Strength training, and so on were included. Detailed characteristics of the included studies are presented in Supplementary Table S1.

### 3.4 Effect of BWSTT on Fugl-Meyer assessment of lower extremity score

**Figure 2:**
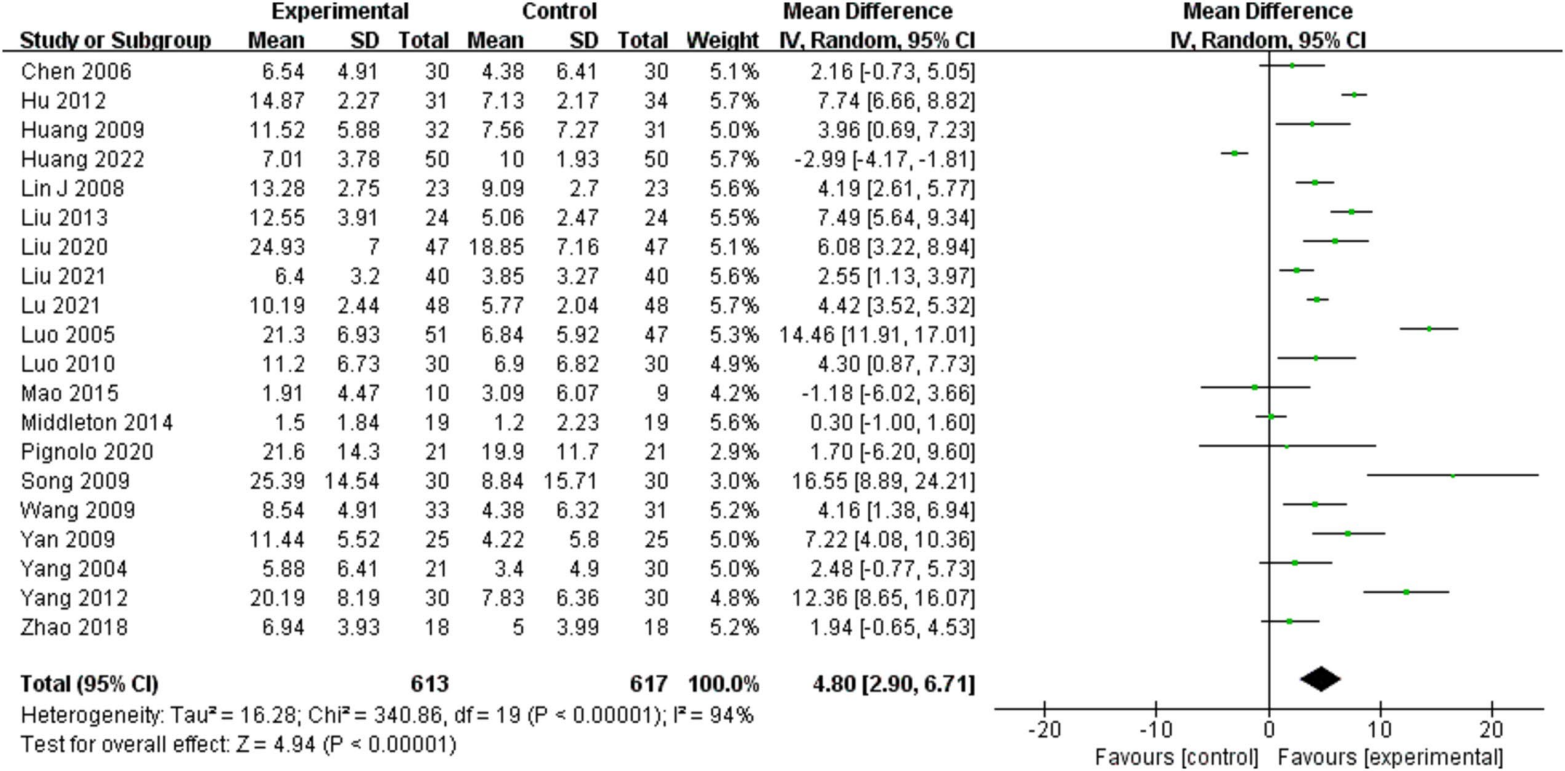
Effect of body weight support training on Fugl-Meyer lower extremity scores.

Fugl-Meyer assessment of lower extremity scores was analyzed for 20 of the 25 included studies (10, 11, 22, 24–29, 31–39, 41, 42). A random effects model was employed for the meta-analysis due to the significant heterogeneity of the combined results (I^2^ = 94%). The combined effect size significantly enhanced the Fugl-Meyer assessment of lower extremity scores for the BWSTT group relative to the control group (MD = 3.60, 95% CI: 1.23–5.98, *p* = 0.003). Figure 2 presents the results.

Four moderating variables were identified to examine the impact of person characteristics and training parameters on the study outcomes: person disease duration, intervention time, maximum body weight support level, and maximum gait speed. The subgroup analysis based on the above moderating variables is shown in Table 1. The results of the study found that the intervention effect was better in persons with a disease duration within the period of 3–6 months (MD = 4.72, 95% CI: 1.54 to 7.89, *p* = 0.004) than in those with a duration of 1–3 months (MD = 4.03, 95% CI: 2.38 to 5.68, *p* < 0.001), while the intervention effect was not statistically significant in person with a disease duration of 6 months or more (MD = -1.36, 95% CI: - 4.58 to 1.87, *p* = 0.41). Intervention time of 4–8 weeks (MD = 5.78, 95% CI: 3.80 to 7.76, *p* < 0.001) was better than 1–4 weeks (MD = 4.25, 95% CI: 1.48 to 7.02, *p* = 0.003), while intervention time of 8 weeks or more (MD = 3.86, 95% CI: −3.18 to 10.91, *p* = 0.28) was not statistically significant. Interventions with a maximum body weight support of 30% or more (MD = 4.51, 95% CI: 1.75 to 7.28, *p* = 0.001) were significantly more effective in improving the Fugl-Meyer assessment of lower extremity score, while interventions with 0–30% (MD = 3.82, 95% CI: −1.49 ~ 9.13, *p* = 0.16) had no statistically significant effect. A maximum gait speed of 0–0.2 m/s or more (MD = 4.01, 95% CI: 1.62 ~ 6.40, *p* = 0.001) significantly improved the effect of the Fugl-Meyer assessment of lower extremity score, while 0–0.2 m/s (MD = 5.68, 95% CI: −0.46 to 11.82, *p* = 0.07) had no statistically significant.

**Table 1:** Subgroup analysis of moderating variables affecting Fugl-Meyer assessment of lower extremity score.

Subgroup	Sample size	Number of studies	Effect size and 95% CI	I^2^ (%)	*P*-value
Disease duration					
1–3 months	468	9	4.03 (2.38, 5.68)	71	<0.001
3–6 months	246	4	4.72 (1.54, 7.89)	87	0.004
More than 6 months	138	2	-1.36(−4.58, 1.87)	94	0.41
Intervention time					
1–4 weeks	453	7	4.25 (1.48, 7.02)	92	0.003
4–8 weeks	471	8	5.78 (3.80, 7.76)	76	<0.001
More than 8 weeks	86	2	3.86(−3.18, 10.91)	97	0.28
Maximum body weight support level					
0–30%	343	5	3.82(−1.49, 9.13)	95	0.16
More than 30%	483	9	4.51 (1.75, 7.28)	95	0.001
Maximum gait speed					
0–0.2 m/s	326	5	5.68(−0.46, 11.82)	98	0.07
More than 0.2 m/s	344	7	4.01 (1.62, 6.40)	79	0.001

As the combined results heterogeneity I^2^ > 50%, the reasons for the heterogeneity were explored by meta-regression analysis (3). The results indicated (Table 2) that maximum body weight support level (*p* = 0.835), intervention time (*p* = 0.249), and maximum gait speed (*p* = 0.266) did not significantly affect heterogeneity. The disease duration (*p* = 0.042) demonstrated a statistically significant result, suggesting that it might have been the main cause of heterogeneity.

**Table 2:** Meta-regression analysis of different moderating variables on Fugl-Meyer assessment of lower extremity score.

Moderating variables	β-regression coefficient	Standard error	t-value	*P*>│t│	95%CI
Disease duration	1.24	0.68	1.82	0.042	(0.248, 2.736)
Intervention time	0.85	0.71	1.2	0.249	(−0.670, 2.379)
Maximum body weight support level	−0.21	0.98	−0.21	0.835	(−2.324, 1.907)
Maximum gait speed	1.30	1.11	1.18	0.266	(−1.165, 3.774)

### 3.5 Effect of BWSTT on Barthel Index score

**Figure 3:**
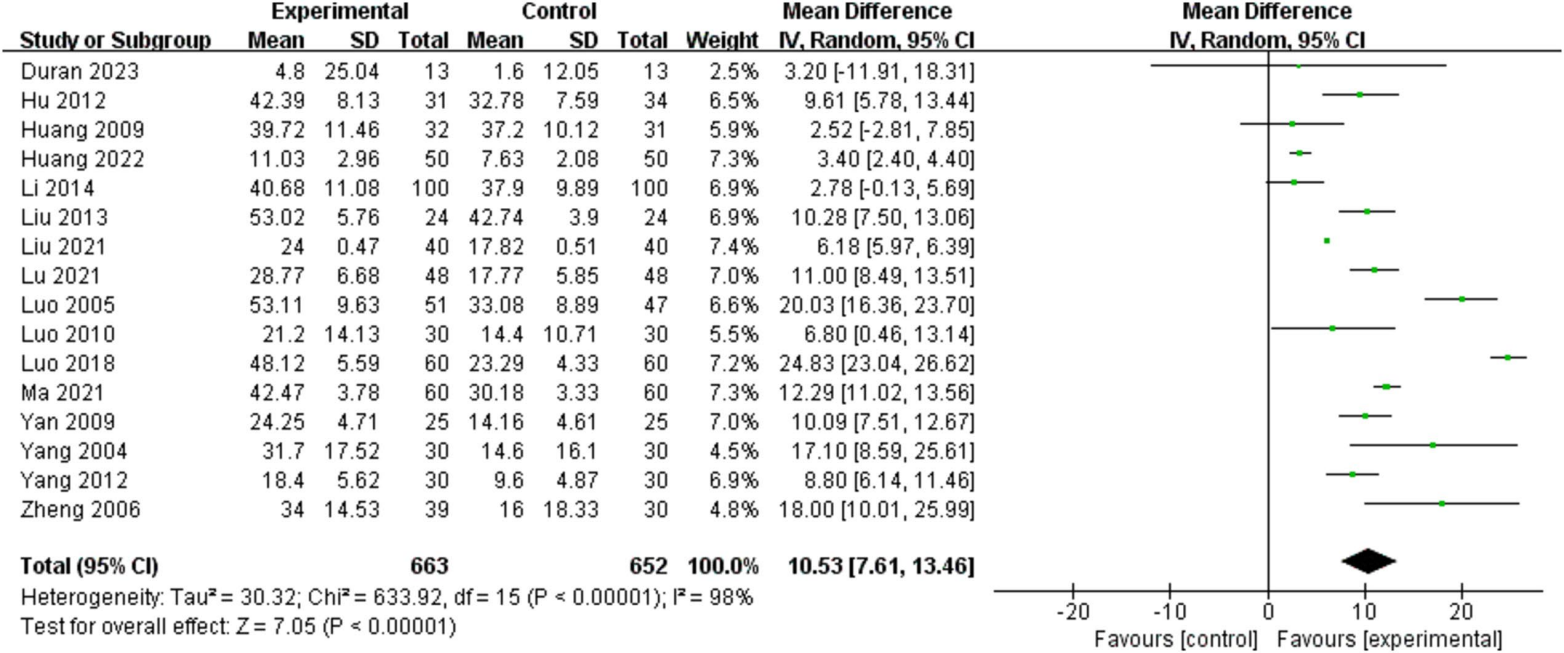
Effect of body weight support training on Barthel Index scores.

Barthel Index scores were analyzed for 16 of the 25 included studies (9, 10, 21–25, 30, 32–35, 37, 40–42). A random effects model was employed for the meta-analysis due to the significant heterogeneity of the combined results (I^2^ = 98%). The effect size indicated a significant enhancement in Barthel Index scores for the BWSTT group relative to the control group (MD = 10.53, 95% CI: 7.61–13.46, *P*<0.001). Figure 3 presents the results.

Subgroup analyses were performed to investigate the influence of pertinent moderating variables on study outcomes. Table 3 presents the subgroup analysis according to the specified moderating variables. The results of the study found that the intervention effect was better in persons with a disease duration within the period of 3–6 months (MD = 17.58, 95% CI: 11.75 to 23.40, *p* < 0.001) than in those with a duration of 1–3 months (MD = 9.65, 95% CI: 4.63 to 14.66, *p* < 0.001) and more than 6 months or more (MD = 3.40, 95% CI: 2.40 to 4.40, *p* < 0.001). Intervention time of 4–8 weeks (MD = 12.85, 95% CI: 3.83 to 21.87, *p* = 0.005) was better than 1–4 weeks (MD = 9.93, 95% CI: 5.86 to 13.99, *p* < 0.001) and more than 8 weeks or more (MD = 8.71, 95% CI: 4.42 to 12.99, *p* < 0.001). Interventions with a maximum body weight support of 30% or more (MD = 10.79, 95% CI: 6.91 to 14.67, *p* < 0.001) were significantly more effective in improving the Barthel Index score, while interventions with 0–30% (MD = 9.36, 95% CI: −1.22 to 19.94, *p* = 0.08) had no statistically significant effect. A maximum gait speed of 0–0.2 m/s or more (MD = 10.61, 95% CI: 1.13 to 20.10, *p* = 0.03) was better than 0–0.2 m/s (MD = 9.08, 95% CI: 2.72 to 15.45, *p* = 0.005).

**Table 3:** Subgroup analysis of moderating variables affecting Barthel Index score.

Subgroup	Sample size	Number of studies	Effect size and 95% CI	I^2^ (%)	*P*-value
Disease duration					
1–3 months	741	8	9.65 (4.63, 14.66)	99	<0.001
3–6 months	129	2	17.58 (11.75, 23.40)	0	<0.001
More than 6 months	126	2	3.40 (2.40, 4.40)	0	<0.001
Intervention time					
1–4 weeks	620	6	9.93 (5.86, 13.99)	97	<0.001
4–8 weeks	358	5	12.85 (3.83, 21.87)	97	0.005
More than 8 weeks	337	5	8.71 (4.42, 12.99)	90	<0.001
Maximum body weight support level					
0–30%	403	5	9.36(−1.22, 19.94)	99	0.08
More than 30%	648	8	10.79 (6.91, 14.67)	92	<0.001
Maximum gait speed					
0–0.2 m/s	210	3	9.08 (2.72, 15.45)	94	0.005
More than 0.2 m/s	572	6	10.61 (1.13, 20.10)	98	0.03

The meta-regression analysis results indicated (Table 4) that disease duration (*p* = 0.065), maximum body weight support level (*p* = 0.761), and maximum gait speed (*p* = 0.439) did not significantly influence heterogeneity. The intervention time (*p* = 0.044) demonstrated a statistically significant result, suggesting that it might be the main cause of heterogeneity.

**Table 4:** Meta-regression analysis of different moderating variables on Barthel Index score.

Moderating variables	β-regression coefficient	Standard error	t-value	*P*>│t│	95%CI
Disease duration	4.45	2.15	2.07	0.065	(−0.34, 9.23)
Intervention time	4.06	1.84	2.21	0.044	(0.12, 7.99)
Maximum body weight support level	1.00	3.23	0.31	0.761	(−6.11, 8.12)
Maximum gait speed	1.55	1.89	0.82	0.439	(−2.92, 6.03)

### 3.6 Sensitivity analyses

Due to the significant heterogeneity among studies, a study-by-study culling approach was employed to evaluate each study’s influence on the overall effect size derived from the collective research (43). The results indicated that the impact on overall heterogeneity after the exclusion of one study was minimal, suggesting the robustness and reliability of the findings for these outcomes (Supplementary Figure S2).

### 3.7 Publication Bias

The funnel plot and Egger’s test were used for the publication bias test. The results showed that the Fugl-Meyer assessment of the lower extremity score funnel plot was more evenly distributed on both sides (Supplementary Figure S3). There was no statistical difference between the test results of Egger’s test (*p* = 0.164, Supplementary Table S2). Still, the Barthel index score funnel plot was unevenly distributed on both sides (Supplementary Figure S3), and there was a statistical difference between Egger’s test results (*p* = 0.002, Supplementary Table S2). Therefore, the Duvaland Tweedie trim and fill method was used to further evaluate the effect of publication bias on the Barthel Index score results. The study results showed that after four iterations and a total of 18 documents after trimming and filling, the amount and significance of the combined effect did not change significantly before and after trimming and filling, indicating that the results of this study were stable. The corrected funnel plot of the supplementary material is shown in Supplementary Figure S4.

### 3.8 Grading of evidence

Using the GRADE tool, the quality of evidence for the included studies was found to be of very low quality (Supplementary Table S3).

## 4 Discussion

Lower limb motor dysfunction and impaired daily living ability are key factors hindering persons with stroke’s self-care and reintegration into family and society (2, 3). This meta-analysis indicates that BWSTT significantly enhances lower limb motor function and daily living ability compared to without BWSTT, consistent with prior research findings (21, 23, 24, 26, 29, 33, 41). The theoretical foundation of BWSTT is rooted in the central pattern generator theory, motor control dynamic systems theory, and the theory of forced use (44). Its mechanism primarily involves high-intensity, task-specific repetitive gait training under reduced weight-bearing conditions. This approach regulates gait speed and body load, facilitating improved lower limb coordination through repetitive pattern generation, enhanced cardiovascular fitness via prolonged aerobic exercise, motor relearning through error correction and sensory feedback, and neural pathway reorganization via central pattern generator activation (44, 45).

**Figure 4:**
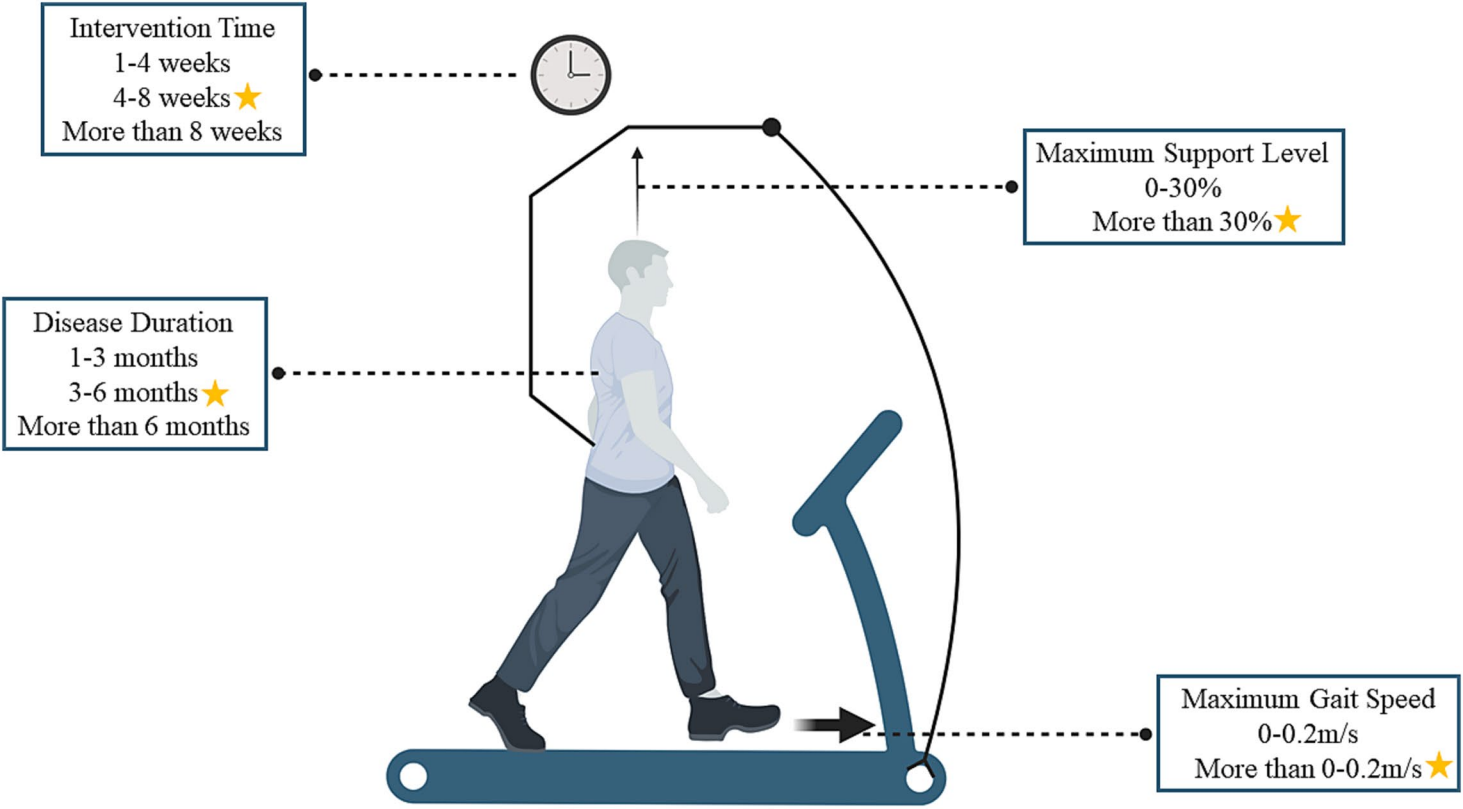
Optimal training strategy for body weight support training (the asterisk indicates the optimal training strategy, using an overhead harness as an example, which also includes pneumatic technology).

Therapists can modify the training load and weight support based on the people’s specific pathology during training, effectively incorporating weight-bearing, stepping, and balance components (3). For individuals unable to train autonomously, BWSTT may improve proprioceptive feedback in the lumbar spinal cord, refine motor neural pathways, and reinforce typical movement patterns (40). Previous research (3) has explored how BWSTT enhances motor function and daily life ability, focusing on motor control, neural pathway transmission, and psychological factors (40). However, from the point of view of the development of rehabilitation training programs, there is a lack of research examining how individual differences in training and variations in training intensity, frequency, and duration affect treatment outcomes. Based on this, the present study explored the optimal training strategy for BWSTT regarding disease duration and training parameters (the study results were subdivided into two aspects: time and load parameters), and the overall findings are shown in Figure 4.

### 4.1 Identifying optimal BWSTT time parameters

Subgroup analyses utilizing moderator variables indicated that an intervention duration of 4–8 weeks yielded optimal outcomes for enhancing lower limb motor function and daily living capabilities in stroke persons with a 3–6 months disease duration. This finding aligns with Jiang et al.’s study on walking function and balance in persons with stroke, further supporting the reliability of the present study (3). Although this study found that persons with a disease duration of 1–3 months who received BWSTT had significantly improved Fugl-Meyer scores (MD = 4.03) and Barthel Index scores (MD = 9.65) compared with the control group, the degree of improvement was lower than that of persons with a disease duration of 3–6 months. Previous studies have suggested that early and appropriate rehabilitation training promotes the regeneration of brain cells around the lesion (46). It is postulated that it induces compensation and reorganization of motor function in the contralateral cerebral hemisphere, accelerating the recovery of lower limb function. Current research generally advocates for early intervention in routine rehabilitation for persons with stroke. However, a study by The AVERT Trial Collaboration group showed that a high-dose, very early mobilization protocol reduces the favorable outcome of persons (47). Meanwhile, a study by Dong et al. on weight support training also suggested that the best outcomes are achieved when the person’s disease duration is less than 1 month. The Lovett unassisted muscle test results were at least grade 2 (as determined by the ability to perform the full range of motion of the joints in a gravity-eliminating position) (48). This may be because, during the acute/subacute early stage (<3 months), the person’s neurological injury status may be more unstable, and inflammatory responses, edema, etc., may affect training tolerance and efficacy (46). Additionally, persons in the early stages may have poorer physical fitness, endurance, and cardiopulmonary function, limiting the maximization of BWSTT training intensity and duration. Therefore, while BWSTT remains an effective rehabilitation modality for persons with a 1–3 month disease course, clinical practice may require more individualized protocols, such as starting with lower intensity and shorter durations, gradually increasing the load, and closely monitoring the person’s tolerance and response. Concurrently, other early rehabilitation strategies should be integrated.

In contrast, the present study found that rehabilitation was most effective for persons with a disease duration of 3–6 months, which differs from Dong et al.’s findings (48). This discrepancy may be due to differences in person inclusion criteria. Dong et al.’s study focused on persons with a disease duration of less than 2 months, not accounting for longer-term persons. In this study, persons in the chronic phase with a disease duration exceeding six months did not show statistically significant improvements in Fugl-Meyer assessment of lower extremity scores. While there was an improvement in the Barthel Index, the magnitude of improvement was minimal (MD = 3.40). Previous studies have suggested that the golden period for neuroplasticity typically occurs within the first 6 months after onset, and the potential for neural remodeling in the chronic phase is relatively reduced, which may lead to a weakened response to training (46, 47). Additionally, persons in the chronic phase may have already developed fixed abnormal movement patterns or compensatory strategies, and altering these patterns may require longer durations and higher intensities of specific training. The standard BWSTT protocol may not break these patterns, affecting training outcomes effectively. Furthermore, the number of studies included in this meta-analysis targeting persons with a disease duration of over 6 months was limited (Fugl-Meyer: *n* = 2; Barthel: *n* = 2), and the sample sizes were relatively small, restricting the statistical power of the results. Therefore, more high-quality studies are needed to confirm the precise efficacy of BWSTT in the chronic phase. This suggests that applying BWSTT in the chronic phase may require combining other intervention methods or adopting higher-intensity, longer-duration, and more personalized BWSTT protocols to address the specific functional impairments and adaptive changes that chronic-phase persons face. Future research should focus on exploring optimized training strategies for BWSTT in chronic stroke patients. Additionally, the results of this study also found that the timing of interventions substantially impacts rehabilitation outcomes. Like this study, Jiang et al. identified 4–8 weeks as the optimal intervention time (3). Unfortunately, the literature lacks additional studies exploring the timing of BWSTT interventions. Future research should validate these findings by including people at different disease stages and using randomized controlled trials to standardize intervention timing.

### 4.2 Identifying optimal BWSTT load parameters

In terms of training load parameters, this study focused on the effects of the maximum body weight support level and maximum gait speed on rehabilitation outcomes. Prior research has yielded inconsistent findings concerning the ideal level of body weight support (3). Jang et al. demonstrated that a body weight support level of 30% or greater was most effective, aligning with the current study’s findings (3). However, Hesse et al.’s study on lower limb EMG suggested that the maximum body weight support level should not exceed 30% (49). This discrepancy may be due to variations in person characteristics. Hesse et al.’s study involved persons with a disease duration of around 40 days and a limited sample size, in contrast to the current study’s wider person range and larger sample size. Further research is needed to clarify these differences.

Regarding the maximum gait speed, Klaske et al. concluded that lower speeds should be avoided during gait training, as they may reduce muscle activation and lead to abnormal gait patterns (50). The present study supports this, showing that a maximum gait speed of 0.2 m/s or more was optimal for improving lower limb motor function and daily living ability in persons with stroke. This indirectly confirms Klaske et al.’s finding that slower speeds are less effective and provide a minimum threshold for gait training speed. However, other studies present different findings. For instance, Wu et al.’s research on different gait speeds indicated that a maximum speed of 0.3 m/s resulted in the most substantial enhancement in motor feedback for persons with stroke (51). In contrast, increased speeds (0.45 m/s) did not improve motor recovery.

Training loads should be tailored to people’s mobility level (3, 52). Persons with lower mobility may benefit from greater body weight support or slower gait speeds, whereas more mobile people may experience diminishing returns from such adjustments. The subgroup analysis of this study did not consider variations in person mobility, and due to the limitations of the included studies, it could not further classify body weight support levels or gait speeds. Future research should investigate the impact of different body weight support ratios and gait training speeds on the rehabilitation of persons with stroke, considering individual mobility variations and appropriately adjusting training loads.

### 4.3 Study limitations

The study presents specific limitations. First, the quality of evidence for the included studies was found to be very low overall, which may compromise the reliability. Second, certain subgroup analyses relied on a restricted number of studies, necessitating further validation of the objectivity of these findings. Future research must emphasize randomized controlled trials with larger sample sizes to improve the rigor of the testing process. Third, this study only focused on BWSTT, which limits the generalizability of the results. Fourth, this study focused on the lower limb motor function and the activities of daily living of persons with stroke. The outcomes mainly used the Fugl-Meyer assessment of lower extremity and the Barthel Index scores. Future studies can use other outcomes to validate the conclusions of this study, according to the purpose of the rehabilitation treatment. Furthermore, conducting more thorough analyses to consider variations in people’s characteristics and interventions based on different device models and support mechanisms of body weight support training (such as an overhead harness or pneumatic) is essential, enhancing the understanding of optimal rehabilitation strategies for persons with stroke. Finally, the high heterogeneity observed in our meta-analysis (I^2^ = 94% for Fugl-Meyer scores; I^2^ = 98% for Barthel Index scores) warrants careful interpretation. We attribute this heterogeneity to methodological (protocol variations, bias risk, and control group heterogeneity) and clinical factors (person disease duration and baseline function). We addressed this via random-effects models, sensitivity analyses, and subgroup analyses (Tables 1–4). However, residual heterogeneity persists. Future trials should standardize protocols using core outcome sets and stratify participants by disease duration to reduce clinical heterogeneity.

## 5 Conclusion

BWSTT demonstrated greater efficacy in improving lower limb motor function and activities of daily living in persons with stroke, with optimal outcomes at disease duration of 3–6 months or undergoing interventions for 4–8 weeks, and more than 30% of the maximum body weight support level or using a gait speed exceeding 0.2 m/s. It is unclear whether persons with disease durations of 3–6 months could achieve the same outcomes as those undergoing 4–8 weeks of intervention. However, due to the limited number and quality of included studies, these conclusions require further validation through high-quality randomized controlled trials.

## Data Availability

The original contributions presented in the study are included in the article/Supplementary material, further inquiries can be directed to the corresponding author.
