# Supplementary material for: Optimal training strategy for body weight support treadmill training to enhance lower limb motor function and activity of daily living in persons with stroke: a systematic review and meta-analysis of randomized controlled trials
Source: Front Neurol. 2025 Sep 2;16:1649246. doi: 10.3389/fneur.2025.1649246 (PMC12436104; doi:10.3389/fneur.2025.1649246)
Supplement: Supplementary file 1 [file Supplementary_file_1.docx]

Supplementary Material

# 1. Supplementary Tables

**Table S1** Characteristics of included studies

| Year | First author | Participants | | |  | Interventions | | | | | | Outcomes |
| --- | --- | --- | --- | --- | --- | --- | --- | --- | --- | --- | --- | --- |
|  |  | Sample size [N (F%)] | Age (Mean±SD) | Disease duration (Days) |  | Device\ Support Mechanism | Intervention time (weeks) | Intervention frequency | Maximum body weight support (%) | Training gait speed (m/s) | Usual rehabilitation training |  |
| 2006 | Zheng[40] | C: 30 (33%) E: 39 (36%) | 51.2±NR | 20-130 |  | NR | Eight to Sixteen | Six times/week, 10-15min/time | 30-60 | 0.25 | Normal limb position, acupuncture, PNF, Stand-up training, et al. | ② |
| 2018 | Zhao[28] | C: 18 (33%) E: 18 (39%) | C: 63.7±9.6 E: 65.3±8.1 | C: 180.3±60.5 E: 150.9±60.1 |  | NR | Four | Five times /week, 30min/time | 30-40 | 0.2-0.4 | Normal limb position, Stand-up training, Activities of daily living training, et al. | ① |
| 2006 | Chen[39] | C: 30 (37%) E: 30 (43%) | C: 59.3±9.6 E: 61.5±10.5 | C: 19.3±11.5 E: 17.1±10.0 |  | Lite Gait\Overhead Harness | Four | Seven times/ week, 5-15min/time | 30 | 0.4 | Normal limb position, PNF, Activities of daily living training, Balance training, et al. | ① |
| 2009 | Wang[11] | C: 13 (NR) E: 33 (NR) | NR | NR |  | NR | Four | NR | NR | NR | Normal limb position, PNF, Balance training, Postural transfer technique, et al. | ① |
| 2009 | Yan[35] | C: 25 (32%) E: 25 (24%) | C: 55.2±10.9 E: 57.6±10.6 | C: 78.8±40.3 E: 80.6±38.5 |  | NR | Six | Six times/week, 5-20min/time | 40-50 | 0.1 | Normal limb position, PNF, Balance training, Postural transfer technique, et al. | ①② |
| 2009 | Song[36] | C: 30 (47%) E: 30 (43%) | C: 58.4±5.8 E: 57.3±5.8 | NR |  | NR | Four | Five times /week, 15-30min/time | 20-40 | 0.09-0.17 | Gait training exercises, et al. | ① |
| 2012 | Yang[33] | E: 30 (43%) C: 30 (47%) | C: 60.5±10.8 E: 61.1±9.8 | NR |  | Lite Gait\Overhead Harness | Twelve | Seven times/week, 30min/time | 30 | 0.2-0.5 | PNF, Balance training, Postural transfer technique, Gait training exercises, et al. | ①② |
| 2018 | Pignolo[27] | E: 21 (33%) C: 21 (24%) | C: 65.6±3.0 E: 66.9±12.0 | C: 14.3±6.6 E: 17.6±15.1 |  | Copernicus® system\Overhead Harness | Six | Five times/week, 120min/time | NR | NR | Passive and active mobilization of lower limbs, Trunk control, standing, Deambulation, et al. | ① |
| 2021 | Ma[23] | C: 60 (38%) E: 60 (35%) | C: 58.4±2.1 E: 58.4±2.1 | C: 19.8±1.6 E: 19.8±1.7 |  | NR | Four | Seven times/week, 20-30min/time | NR | NR | Normal limb position, Balance training, Activities of daily living training, et al. | ② |
| 2021 | Lu[24] | C: 48 (42%) E: 48 (38%) | C: 62.9±3.5 E: 63.0±3.5 | NR |  | NR | One to Three | Three times/week, 20-30min/time | NR | NR | Gait training exercises, Stand-up training, Activities of daily living training, et al. | ①② |
| 2021 | Liu[25] | C: 40 (38%) E: 40 (35%) | C: 52.1±13.4 E: 53.1±12.8 | C: 12.7±4.2 E: 11.2±4.6 |  | NR | Four | Six times/week, 30min/time | 35 | 0.15-0.45 | PNF, Balance training, Gait training exercises, Stand-up training, Muscle strength training, et al. | ①② |
| 2013 | Liu[32] | C: 24 (29%) E: 24 (25%) | C: 56.1±6.0 E: 55.2±7.1 | C: 60.7±6.0 E: 61.4±7.6 |  | NR | Twelve | Five times/week, 15min/time | NR | NR | Brunnstrom, Bobath, PNF, Balance training, Gait training exercises, et al. | ①② |
| 2020 | Liu[26] | C: 47 (47%) E: 47 (43%) | C: 58.7±4.1 E: 59.0±4.6 | C: 90.5±30.5 E: 90.7±30.4 |  | NR | Eight | Seven times/week, 30min/time | NR | NR | Stand-up training, Gait training exercises, Postural transfer technique, et al. | ① |
| 2004 | Yang[42] | C: 30 (30%) E: 21 (29%) | C: 55.2±10.7 E: 54.0±10.7 | C: 127.6±68.3 E: 130.3±64.5 |  | NR | Six | Five times/week, 30min/time | 30-40 | 0.14 | Physical therapy | ①② |
| 2008 | Lin[38] | C: 23 (30%) E: 23 (35%) | C: 53.6±10.2 E: 51.3±10.8 | C: 28.7±16.7 E: 30.5±15.3 |  | NR\Overhead Harness | Four to six | Five times/week, 10-30min/time | 40 | 0.2-2 | Gait training exercises, Postural transfer technique, Acupuncture, et al. | ① |
| 2014 | Li[30] | C: 100 (44%) E: 100 (42%) | C: 61.3±8.9 E: 59.4±9.7 | C: 18.1±9.0 E: 16.4±9.3 |  | NR\Overhead Harness | Four | Five times/week, 15-20min/time | 40 | 0.2-0.4 | Brunnstrom, Bobath, PNF, et al. | ② |
| 2009 | Huang[37] | C: 31 (35%) E: 32 (44%) | C: 58.3±13.4 E: 60.5±11.3 | C: 16.5±9.7 E: 15.3±10.4 |  | NR | Six | Seven times/week, 15-20min/time | 30 | 0.5 | Muscle strength training, Gait training exercises, Postural transfer technique, Balance training, et al. | ①② |
| 2022 | Huang[22] | C: 50 (44%) E: 50 (38%) | C: 53.1±8.2 E: 49.3±5.2 | C: 720.5±360.7 E: 720.7±360.5 |  | NR\Overhead Harness | Twelve | Five times/week, more than 20min/time | 30 | 0.15 | Muscle strength training, PNF, Gait training exercises, Balance training, et al. | ①② |
| 2012 | Hu[34] | C: 34 (38%) E: 31 (39%) | C: 61.4±8.5 E: 62.8±7.3 | NR |  | NR\Overhead Harness | Eight | Five times/week, 15-30min/time | 30-40 | NR | Normal limb position, Gait training exercises | ①② |
| 2005 | Luo[41] | C: 47 (57%) E: 51 (51%) | C: 55.0±6.9 E: 53.0±8.7 | NR |  | Lite Gait\Overhead Harness | Four | Seven times/week, 15-30min/time | 60 | 0.1-0.3 | Brunnstrom, Bobath, PNF, Gait training exercises, Balance training, et al. | ①② |
| 2014 | Middleton[31] | C: 20 (20%) E: 23 (39%) | C: 60.7±14.4 E: 61.4±15.7 | C: 870.0±690.9 E: 1500.4±1680.8 |  | NR | Ten | NR | 8-50 | NR | Balance, strength and coordination activities, ROM, Overground gait training | ① |
| 2015 | Mao[29] | C: 9 (78%) E: 10 (80%) | C: 60.8±10.7 E: 59.6±9.2 | C: 47.7±16.8 E: 49.3±19.5 |  | Noramco  Fitness and SpinoFlex\Overhead Harness | Three | NR | 30-40 | 0.5-2.5 | Gait training exercises, et al. | ① |
| 2010 | Luo[10] | C: 30 (43%) E: 30 (37%) | C: 61.5±10.5 E: 59.3±9.6 | C: 30.1±10.0 E: 32.3±11.5 |  | Lite Gait\Overhead Harness | Sixteen | Seven times/week, 5-15min/times | 30 | 0.4 | PNF, Gait training exercises, Balance training, et al. | ①② |
| 2023 | Duran[21] | C: 13 (NR) E: 13 (NR) | C: 57.9±10.9 E: 54.1±18.9 | C: 300.0±150.1 E: 360.0±120.0 |  | AlterG\Pneumatic | Four | Three times/week, 5-15min/time | 65-100 | 0-0.1 | Muscle strength training, Gait training exercises, Normal limb position, et al. | ② |
| 2018 | Luo[9] | C:60 (40%) E:60 (45%) | C: 61.7±8.2 E: 60.7±5.9 | C: 15.4±10.4 E: 16.1±9.9 |  | Lite Gait\Overhead Harness | Six | Seven times/week, 20min/time | 30 | 0.5 | Muscle strength training, Gait training exercises, Normal limb position, Balance training, et al. | ② |

E, experimental group; C, control group; NR, not reported; PNF, proprioceptive neuromuscular facilitation; ①, Fugl-Meyer assessment of lower extremity score; ②, Barthel Index score.

**Table S2** Results of the Egger's test

| Outcomes | Std_Eff | Coef. | Std.Err. | t | *P*＞│t│ | 95%CI |
| --- | --- | --- | --- | --- | --- | --- |
| Fugl-Meyer assessment of lower extremity score | slope | -0.79 | 1.17 | -0.68 | 0.506 | (-3.25，1.67) |
|  | bias | 6.01 | 4.13 | 1.45 | 0.164 | (-2.71，14.73) |
| Barthel Index scores | slope | -1.43 | 0.75 | -1.89 | 0.079 | (-3.04，0.19) |
|  | bias | 10.98 | 2.84 | 3.87 | 0.002 | (4.89，17.07) |

**Table S3** Assessment of quality of evidence

| Certainty assessment | | | | | | | |
| --- | --- | --- | --- | --- | --- | --- | --- |
| Outcomes | Participants (studies) | Risk of bias | Inconsistency | Indirectness | Imprecision | Publication bias | Overall certainty of evidence |
| Fugl-Meyer assessment of lower extremity score | 1230 (20 RCTs) | Very serious^a^ | Serious^b^ | Not serious | Serious^c^ | None | ⨁○○○  Very low |
| Barthel Index scores | 1315 (16 RCTs) | Very serious^d^ | Serious^b^ | Not serious | Serious^c^ | None | ⨁○○○  Very low |

a, sixteen of the included studies were ranked as High risk of bias; b, substantial heterogeneity, 95% CI do not entirely overlap; c, small number of events with rather wide 95% CI; d, fourteen of the included studies were ranked as High risk of bias.

# 2. Supplementary Figures


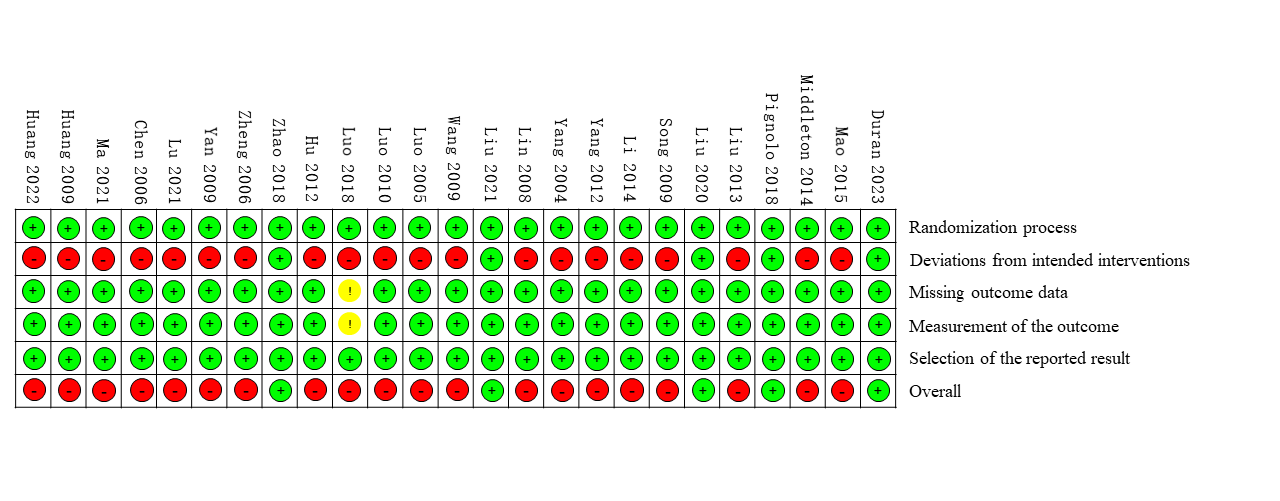


**Fig. S1** Results of quality evaluation of included literature


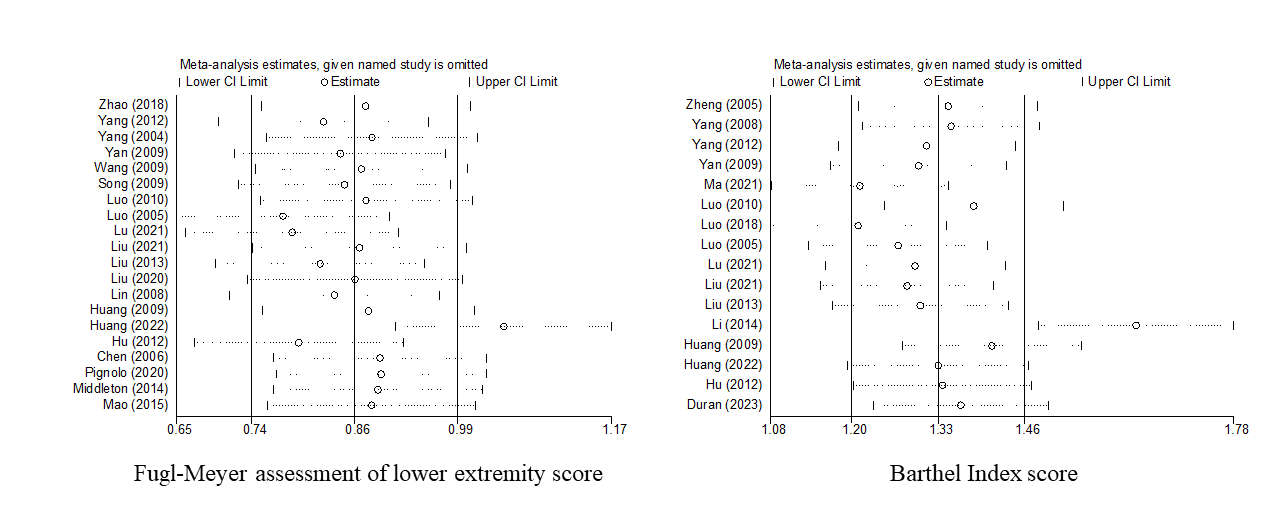


**Fig. S2** Sensitivity analysis


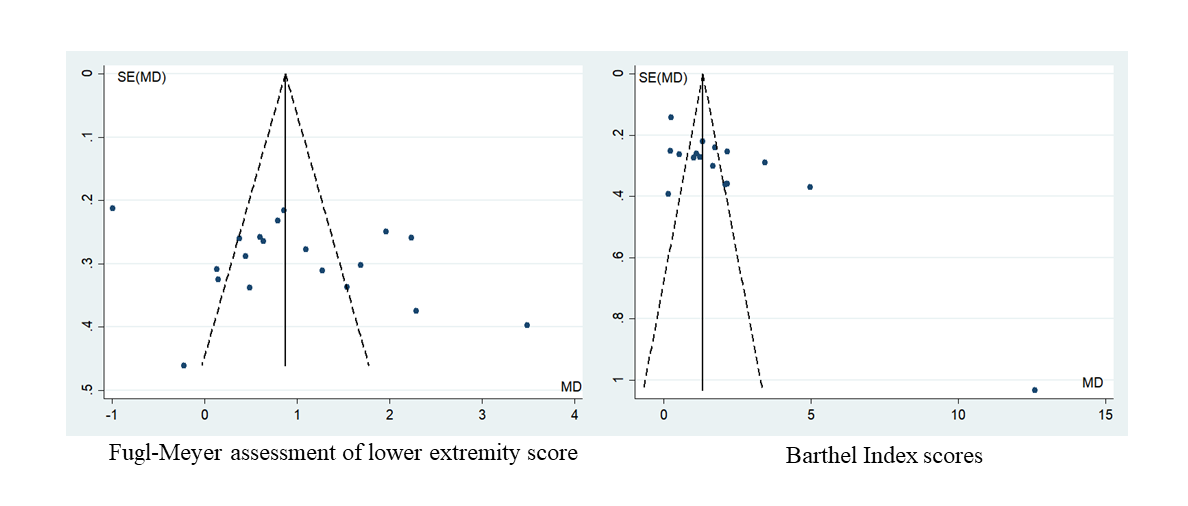


**Fig. S3** Meta-analysis publication bias funnel plot


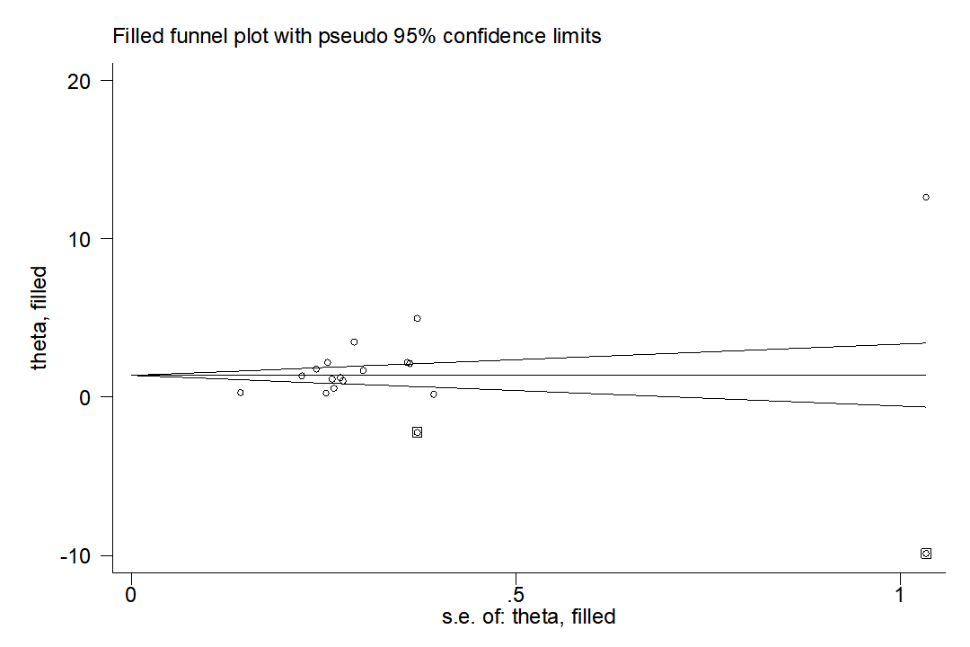


**Fig. S4** Barthel Index scores funnel plot after correction by trimming and filling
